# Enterprise stent for the treatment of symptomatic intracranial atherosclerotic stenosis: an initial experience of 44 patients

**DOI:** 10.1186/s12883-015-0443-9

**Published:** 2015-10-08

**Authors:** Zhengzhe Feng, Guoli Duan, Ping Zhang, Lei Chen, Yi Xu, Bo Hong, Wenyuan Zhao, Jianmin Liu, Qinghai Huang

**Affiliations:** Department of Neurosurgery, Changhai Hospital, Second Military Medical University, 168 Changhai Rd, Shanghai, 200433 China

**Keywords:** Atherosclerosis, Endovascular, Enterprise, Intracranial stenosis, Stent

## Abstract

**Background:**

Wingspan stenting for the treatment of complex intracranial atherosclerotic stenosis (ICAS), i.e., that involving tortuous vascular pathways, long (>15 mm) lesions or arterial bifurcations, has a relatively high risk of complications. This retrospective study assessed the safety and efficacy of undersized balloon angioplasty followed by deployment of the more flexible Enterprise stent for the treatment of complex symptomatic ICAS.

**Methods:**

Forty-four patients on combined antiplatelet therapy and intensive risk factor management and a symptomatic 70–99 % stenosis of a major intracranial artery in complex settings that was treated with balloon angioplasty and Enterprise stent deployment between July 2009 and August 2013 were enrolled. Primary outcome was occurrence of ischemic or hemorrhagic stroke or death within 30 days after intervention. Secondary outcomes included procedural success (defined as achievement of <50 % immediate residual stenosis), and follow-up clinical and angiographic outcomes.

**Results:**

With a procedural success rate of 100 %, stenosis was reduced from 79.3 ± 8.1–14.9 ± 12.3 %. Three (6.8 %) ischemic and 1 (2.2 %) hemorrhagic strokes occurred during the periprocedural period, with no further transient ischemic attacks or strokes in the 42 patients available at median 25.6 (range, 12–57) months follow-up. Of the 38 (86.4 %) patients who underwent angiographic follow-up, 3 (6.81 %) developed >50 % in-stent restenosis after mean 22 months follow-up.

**Conclusion:**

In this retrospective, single-center experience, undersized balloon angioplasty followed by Enterprise stent deployment appears technically feasible with a relatively low rate of complications for the treatment of complex symptomatic ICAS. Prospective, multicenter, randomized controlled trials against optimal medical management are warranted.

## Background

Approximately 5–10 % of all strokes and transient ischemic attacks (TIAs) are secondary to intracranial atherosclerotic disease (ICAD), whose incidence varies by race being more prevalent among Chinese, Japanese, African-Americans, and Hispanics [1, 2]. Although endovascular treatment emerged in the 1980s as a potential option for intracranial atherosclerotic stenosis (ICAS) with promising results upon device development and improvement [3, 4], its superiority to optimal medical treatment remains controversial [5]. In SAMMPRIS, the only randomized controlled trial (RCT) comparing the Wingspan stent and optimal drug therapy, Wingspan stent use was associated with a higher rate of perioperative complications [6]. Complex ICAS, i.e., that in tortuous vascular segments, long (>15 mm) lesions and/or arterial bifurcations, has been associated with higher rates of complications and renders particularly difficult the deployment of the Wingspan stent because of its high radial force and rigidity [7, 8]. The self-expanding, closed-cell Enterprise stent, which was originally designed for neck remodeling in the treatment of wide-necked intracranial aneurysms [9], appears better suited than the Wingspan stent for the treatment of complex ICAS because of its high flexibility, special carrier system structure [10] and reduced radial force [10], which is nonetheless sufficient to prevent elastic recoil and restenosis. In the study by Henkes et al. [11], treatment of intracranial atherosclerotic arterial stenosis with the Enterprise stent yielded a relatively lower in-stent restenosis rate than with the Wingspan stent; however, reperfusion hemorrhage and plaque shift in high-grade (>70 %) stenoses remained concerns. This paper reports the long-term safety and efficacy results of our single-center experience since we began in 2009 to use balloon dilatation and Enterprise stent deployment exclusively for the treatment of complex severe ICAS.

## Methods

Written informed consent was obtained from all participants. In addition, our local institutional ethics review board (the Medical Ethics Committee of Second Military Medical University) approved the research protocol for the retrospective study. Participants of the current study also agreed with publication of their personal information for research purposes.

### Patient population

From July 2009 to August 2013, 357 patients whose CTA/MRA confirmed severe stenosis (>70 %) were admitted to our department for further diagnostic cerebral angiography. Among them, 298 patients demonstrated severe stenosis (>70 %) and were treated by percutaneous transluminal angioplasty and stenting (PTAS: 34 with the Solitaire stent; 137 with the Wingspan stent; and 83 with domestic stents). Of the 298 patients, 44 patients were treated with the Enterprise stent and met the following inclusion criteria: (1) stenosis >70 % as determined by digital subtraction angiography (DSA) using the formulas described by the Warfarin Aspirin Symptomatic Intracranial Disease (WASID) method; (2) recurrent low-flow TIAs or non-disabling ischemic stroke despite antiplatelet or anticoagulation therapy (at our center, patients with symptomatic ICAS undergo close outpatient follow-up and aggressive medical management according to the guidelines for the prevention of stroke in patients with stroke and TIA); and (3) stenoses located in lesions that are long, at a bifurcation, and/or with tortuous access, and therefore particularly challenging for Wingspan stent use because of its rigidity. Patients with any of the following were excluded: (1) total occlusive lesion; (2) severe disability because of stroke or dementia; or (3) inability to give informed consent.

### Lesion characteristics

All patients underwent cerebral angiography to confirm the diagnosis, and three-dimensional reconstruction was performed to accurately measure the morphology and access of the targeted segment of stenosis. The measurement of normal and stenotic vessel diameter was performed using the two-dimensional angiographic image with the help of a measuring ball, and the degree of stenosis was calculated with the formulas described by the WASID method [12]. We used the LMA classification [13] that Jiang et al. described to identify the location, morphology, and access of intracranial stenosis. Among the 44 lesions studied, 24 were located in the middle cerebral artery, one in the anterior cerebral artery, 8 in the intracerebral vertebral artery (VA), and 11 in the basilar artery trunk. The average vascular lesion length was 11.64 ± 6.33 mm (range, 4.20–28.00), and the mean reference artery diameter was 2.63 ± 0.79 mm (range, 1.52–4.01). Stenosis was located adjacent to bifurcations in 28 (63.6 %) patients.

### Procedures

A dose of 300 mg of acetylsalicylic acid (ASA) and 75 mg of Clopidogrel was given at least 3 days before endovascular treatment, which was always performed under general anesthesia. A 6 F guiding catheter was introduced through a femoral sheath. During intervention, patients were heparinized to a doubled activated clotting time. A radiologic examination of the target vessel was performed using a biplane angiographic system (Artis zee Biplane; Siemens, Erlangen, Germany) or a single-arm angiosuite (Philips FD, Netherlands). Rotational angiography followed by 3D reconstruction was performed to understand the normal vessel diameter adjacent to the stenosis and the diameter and the length of the stenosis. The enhanced Dyna CT was also performed to access the location of the plaque and the relationship between the stenosis and important vessel branches. Under a road-map, the vessel distal to the stenosis was catheterized with Transcend Floppy microguidewire (Boston, Scientific), followed by predilatation with a balloon (Gateway, Stryker). After deflation and withdrawal of the balloon catheter, a flushed 0.021-inch microcatheter (Prowler Select Plus, Codman) was navigated over the exchange-wire. The Enterprise stent (Codman) was deployed to cover the targeted stenosis. A daily dose of aspirin (300 mg) and clopidogrel (75 mg) was recommended for 6 weeks after discharge followed by only aspirin. We did not perform routine response testing for aspirin or clopidogrel mainly because of cost and coverage constraints. Treatment for hypertension, and to normalize blood glucose and lipid levels if needed was also suggested. All patients received statin therapy after stenting, 30 (68.1 %) patients with hypertension were treated with antihypertensive drugs, and 13 (29.5 %) patients with diabetes mellitus received either metformin or insulin therapy.

### Evaluation of outcomes and clinical and angiographic follow-up

The primary outcome for this study was occurrence of any stroke or death during/after the procedure and at 30-day follow-up. Secondary outcomes were procedural success, occurrence of >50 % in-stent restenosis confirmed by DSA, and occurrence of transient ischemic attack (TIA) or stroke attributable to the target vessel during the follow-up observation period.

Procedural success was defined as accurate delivery and deployment of the stent (in all intended stenting procedures), covering the target lesion completely and resulting in a residual stenosis of less than 50 %.

The neurological status was documented according to the National Institute of Health Stroke Scale (NIHSS) score at discharge. A TIA was defined as neurological worsening with complete recovery within 24 h. Minor and major stroke was defined as persistent worsening of the NIHSS score of <4 or ≥ 4 points, respectively. We defined symptomatic brain hemorrhage as parenchymal, subarachnoid, or intraventricular hemorrhage detected by CT or MRI that was associated with new neurological signs or symptoms lasting 24 h or more or a seizure. The initial and follow-up clinical evaluations were performed by two neurologists (P Zhang and L Chen).

Angiographic follow-up was scheduled at 3 and 6 months and yearly thereafter with DSA, CTA, and/or CTA/CTP. DSA was routinely used to assess restenosis, and although image might be affected by stent-related artifacts, CTA was used when patients refused invasive assessment. ISR was defined as >50 % stenosis within or immediately adjacent (within 5 mm) to the implanted stent with >20 % absolute luminal loss in 2D-DSA image.

### Statistical methods

Statistical analyses were performed using SAS 9.1 (SAS Institute Inc., Cary, NC). Mean, median, and standard deviation were used for descriptive statistics. Differences between before and after treatment were analyzed using the t- and chi-square tests for parametric and nonparametric data, respectively. *P* < 0.05 was considered statistically significant.

## Results

### Patient characteristics

The study cohort included 44 patients (32 men and 12 women) with a mean age of 60.45 ± 9.07 years (range 36–78 years). All patients presented with an ischemic stroke (26 patients, 59.09 %) or TIA (18 patients, 40.91 %) in the territory of a suspected 70–99 % stenotic intracranial artery. Distal ischemia was present in 12 patients, and hemodynamic compromise in 32 patients; there were no cases of perforator stroke, which might have increased procedural risk. The stenting procedure was performed 43.40 ± 10.37 days (range 9–67 days) from the qualifying event. The general characteristics of the included patients are summarized in Table 1.

**Table 1:** Demographic and clinical characteristics of the 44 patients treated with Enterprise stents for complex symptomatic intracranial stenosis

	*N* (%)
Age, years	60.45 ± 9.07
Male gender	32 (72.73)
Symptoms	
TIA and other ischemic events	18 (40.91)
Stroke	26 (59.09)
Hypertension	30 (68.18)
Hyperlipidemia	9 (20.45)
Smoking	11 (25.00)
Diabetes mellitus	13 (29.55)
Coronary artery disease	2 (4.55)
Body mass index	
18.5–23.9	16 (36.36)
24 ~ 27.9	24 (54.55)
≥28	4 (9.09)
NIHSS score before intervention	
1	14 (31.82)
2	19(43.18)
3	11 (25.00)
Time intervals from last event to intervention, day	
<30	11 (25.00)
≥30	33 (75.00)
Symptom pattern unstable/stable	1/43

Note: *TIA* transient ischemic attack, Symptom pattern unstable indicates resolved, improving or unchanging symptoms before the stent placement procedure; unstable indicates progressive or fluctuating neurologic symptoms (National Institutes of Health Stroke Scale score > 4) corresponding to intracranial stenosis within the 2 days prior to the stent placement procedure

### Lesion and angiographic characteristics

All 44 patients underwent digital subtraction angiography (DSA) preoperatively. The degree of stenosis was determined by using the formulas described by the WASID method. The distal artery diameter was defined as the reference artery diameter. Lesion classification was according to the morphology and access criteria system, as described by Jiang et al. The lesion characteristics are summarized in Table 2.

**Table 2:** Lesion characteristics for the 44 patients treated with Enterprise stents for complex symptomatic intracranial stenosis

Characteristics	*N* (%)
Location (i)	
Anterior circulation	25 (56.82)
Posterior circulation	19 (43.18)
Location (ii)	
MCA	24(54.54)
ACA	1 (2.27)
PCA	0 (0.00)
BA	11 (25.00)
VA	8 (18.18)
Stenosis grade, %	
70–79	18 (40.90)
80–89	13 (29.55)
90–99	13 (29.55)
Mean stenosis degree, %	70–92 (mean, 79.32 ± 8.18)
Vascular lesion length, mm	4.20–28.00 (mean, 11.64 ± 6.33)
Reference artery diameter, mm	1.52–4.01 (mean, 2.63 ± 0.79)
Classification of morphology	
A	4 (9.09)
B	21 (47.73)
C	19 (43.18)
Classification of access	
I	22 (50.00)
II	17 (38.64)
III	5 (11.36)

Note: *MCA* middle cerebral artery, *ACA* anterior cerebral artery, *PCA* posterior cerebral artery, *VA* vertebral artery, *BA* basilar artery, Classification was according to the morphology and access criteria system

### Procedural results and periprocedural complications

The overall procedural success rate was 100 %. The average degree of target stenosis before treatment, after balloon dilatation and after stent deployment was determined as 79.3 ± 8.1, 23.4 ± 9.5 % and 14.9 ± 12.3 %, respectively.

Thirty-day follow-up was available for all 44 patients. Major procedure-related complications (stroke or death) during the periprocedural period (30 days) were encountered in 4 (9.1 %) cases. Three patients developed a new emerging ischemic stroke that was classified as a perforator event, including two upper basilar trunks and one M1 segment. One patient experienced intraparenchymal hemorrhage due to hyperperfusion injury. At our department, blood pressure is strictly controlled after intervention in all patients to avoid hyperperfusion injury. Two patients developed groin hematoma after the procedure and recovered after hemostasis by compression; one patient developed pneumonia because he was bedridden. The lesion characteristics of the latter four patients who suffered complications are summarized in Table 3. No patient suffered myocardial infarction, gastrointestinal bleeding, or died during the periprocedural period.

**Table 3:** Lesion characteristics and treatment results of 4 patients suffering perioperative complications

Case no.	Location	LMA classification	Balloon size (mm)	Stent size (mm)	Pre/Post stent stenosis (%)	Time interval (after-procedural)	MR image	Symptoms	NIHSS
1	MCA	T/A, C, II	1.5/9	4.5/28	90/40	3 h	Infarction	Decreased muscle strength	2
2	MCA	B/B, B, II	2.25/9	4.5/22	90/0	5 h	ICH	Hemiplegia	2
3	BA	T/N, C, I	1.5/9	4.5/22	75/0	40 h	Infarction	Visual field defects	1
4	BA	T/N, C, II	1.5/15	4.5/22	70/20	40 h	Infarction	Decreased muscle strength	1

Record of LMA classification. Situation: origin (O) or trunk (T) or bifurcation (B)/location, morphology, access classification. *ICH* intracranial hemorrhage

### Clinical and angiographic follow-up results

A clinical evaluation was performed in all patients at discharge using the National Institute of Health Stroke Scale (NIHSS) score. The NIHSS score at discharge was 0 in 25 patients, one in 17 patients and two in 2 patients.

**Fig. 1:**
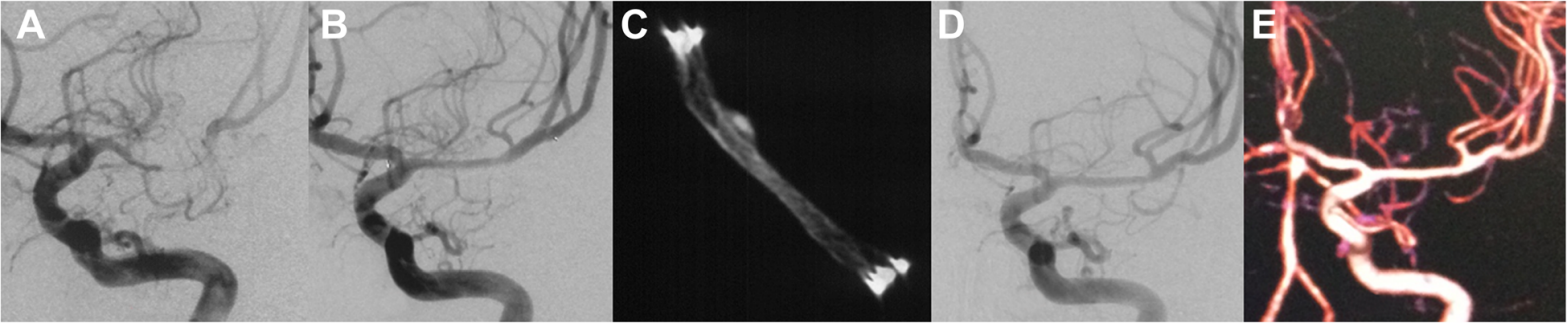
A 41-year-old man with a severe stenosis of the M1 segment of the left middle cerebral artery (*MCA*) was referred for evaluation of stroke. Diagnostic cerebral angiography confirmed a preocclusive (>80 %) stenosis of the left MCA **a**. The patient underwent percutaneous transluminal angioplasty and Enterprise stent placement. The subtracted image demonstrates near 30 % postprocedural residual stenosis **b**. The DynaCT scan shows the morphology of the stent after placement **c**. The 11 months follow-up angiogram shows complete patency of the stented segment **d** and good outcome at 30 months follow-up as confirmed by CTA **e**

**Fig. 2:**
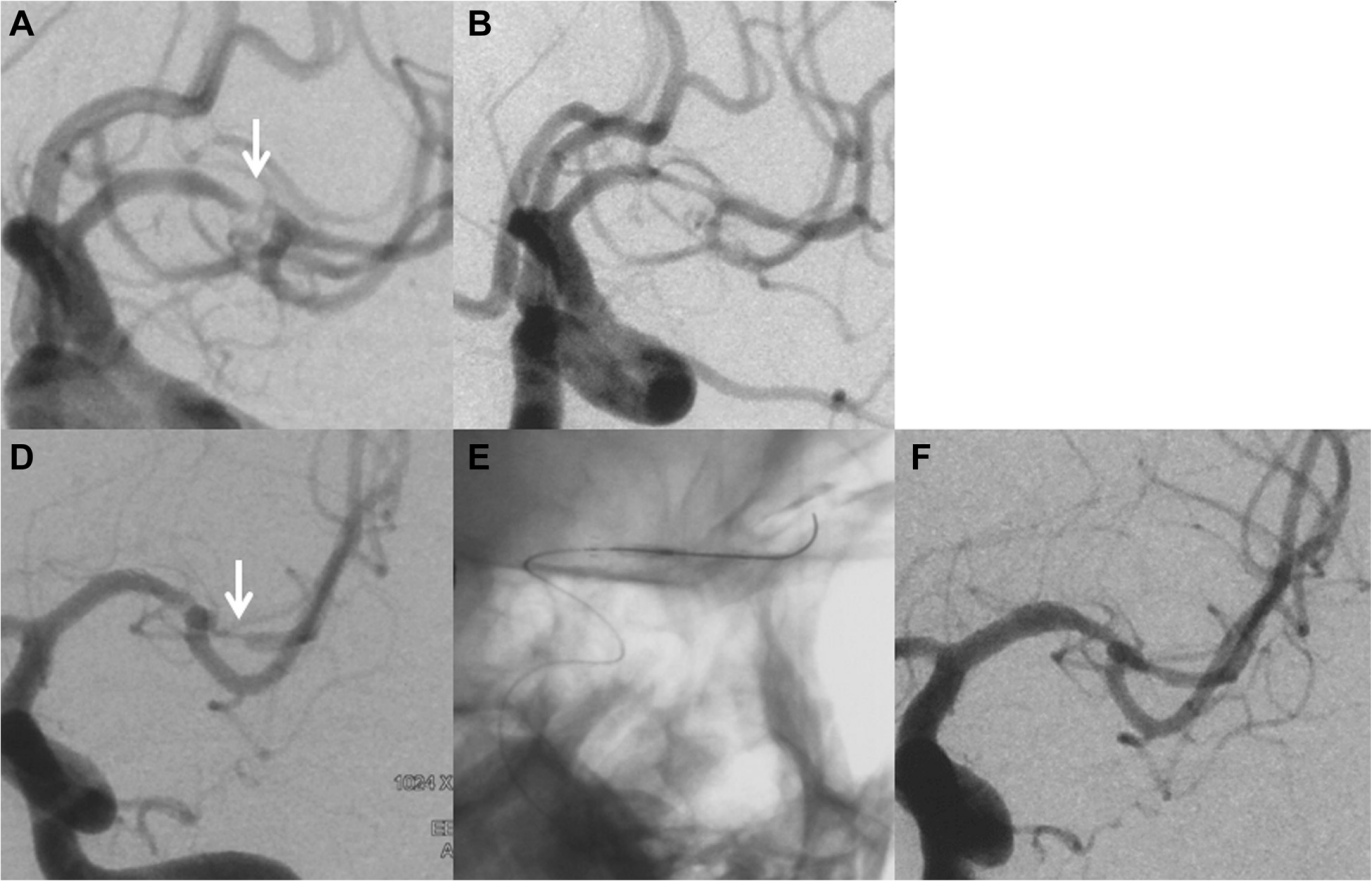
A 39-year-old man with a severe stenosis of the M1 segment of the left middle cerebral artery (*MCA*) was referred for evaluation of stroke. Diagnostic cerebral angiography confirmed a preocclusive (90 %) stenosis of the left MCA **a**. The patient underwent percutaneous transluminal angioplasty and Enterprise stent placement. The subtracted image demonstrates near 30 % postprocedural residual stenosis **b**. After 4 months, the patient experienced TIAs in the dependent territory, and follow-up angiography revealed in-stent restenosis (80 %) and diffuse intimal hyperplasia throughout the entire stented segment **d**. Angioplasty was performed with a slow, graded inflation of the balloon (Gateway, 1.5/9) to a pressure of 7 atm **e**. The subtracted image post retreatment demonstrates near 30 % residual stenosis **f**

A total of 44 patients were available for a clinical follow-up visit 12–57 months (median 25.6 months) after treatment. Thirty-one patients had their follow-up visit more than 24 months after intervention (Fig. 1). No patient experienced recurrent TIA or stroke or died. Overall, 38 patients (86.3 %) underwent DSA follow-up examinations and another 6 patients underwent CT angiography during the follow-up period. The median angiographic follow-up period was 11.5 months (6–30.2 months). Three patients (6.8 %) developed ISR, defined as an in-stent stenotic lesion of more than 50 % on the follow-up DSA series, after an average follow-up of 22 months. The lesion characteristics of the three patients who developed ISR are summarized in Table 4. One (2.3 %) of these recurrent lesions received balloon angioplasty (Pre/Post retreatment stenosis, 80/30 %) (Fig. 2).

**Table 4:** Lesion characteristics and treatment results of 3 patients with IRS at follow-up visit

Case no.	Location	LMA classification	Length of lesion (mm)	Balloon size (mm)	Balloon pressure (atm)	Stent size (mm)	Pre/Post stent stenosis (%)	Follow-up (DSA) (months)	ISR (%)	Symptoms	Retreatment
1	MCA	B/C, B, II	7.22	2/15	5.0	4.5/22	70/10	29	55	Decreased muscle strength	No
2	MCA	B/A, B, II	7.11	1.5/9	6.0	4.5/14	90/30	5	80	Decreased muscle strength strength	Balloon angioplasty (residual stenosis 30 %)
3	BA	T/N, B, I	9.75	2.5/15	5.0	4.5/15	90/30	7	70	None	No

## Discussion

In this retrospective single-center study, undersized balloon angioplasty followed by stenting with the Enterprise stent appeared safe and effective in treating complex symptomatic ICAS, i.e., that in tortuous vascular segments, long lesions or bifurcations which render challenging deployment of the Wingspan stent and are associated with higher periprocedural risk rates. Use of the Enterpise stent in the latter setting was associated with a higher procedural success rate (100 %), lower rate of complications (perioperative stroke incidence, 9.1 %) and better long-term outcome (ISR rate, 6.8 %) than published results for the Wingspan stent.

The best treatment option for symptomatic intracranial atherosclerotic stenosis remains controversial [14, 15]. Results from WASID and SAMPPRIS studies suggested that optimal medical therapy may reduce the recurrence rate of stroke [6, 16]. Intracranial stents are also thought to minimize stroke recurrence by improving blood flow, especially for severe stenosis with serious hypoperfusion [17, 18]. Since 2000, many studies have reported the safety and efficacy of stenting for ICAD. Wingspan is currently the only self-expanding stent approved by FDA for ICAD treatment. Many single- and multicenter clinical studies have proven that stenting for ICAD is relatively safe, however, in the multicenter, randomized controlled SAMMPRIS trial, optimized medical therapy was superior to stenting combined with medical treatment in prevention of stroke for patients with ICAD.

Possible explanations for the higher periprocedural event rate in SAMMPRIS than in previous registries include higher stenosis severity and earlier treatment in SAMMPRIS (within 30 days of the qualifying event) [11, 19]. The design characteristics of the Wingspan stent also increased procedural difficulties and risks, particularly in tortuous vascular pathways, longer-segment lesions or cases involving arterial bifurcation [20–22]. The LMA classification seems to be helpful in informing individual therapy and predicting stenting results. In cases with tortuous access pathways, it became difficult and dangerous to superselect or navigate the Wingspan system to the target the stenosis segment. Jiang and his colleagues reported a mortality rate of 0, 0, and 25 % for types A, B, and C lesions, respectively [13]. In this study, 21 patients (47.7 %) and 19 patients (43.1 %) were classified with type B and C morphology, respectively. All procedures were performed successfully without fatalities. According to LMA classification, 17 (38.6 %) and 5 (11.3 %) patients were classified as type II and III, respectively, by access. The high procedural success rate might be related with the design of the stent delivery system. The Enterprise self-expanding nitinol stent was originally developed for neck remodeling in the treatment of wide-necked intracranial aneurysms. The stent is introduced through a standard 0.021-inch inside diameter microcatheter and can be partially deployed as much as 70 % within the artery with the possibility of recapturing and redeployment if needed [23].

In a previous study, Henkes and his colleagues demonstrated that undersized balloon angioplasty and deployment of an Enterprise stent is safe and effective for intracranial stenosis, resulting in a rate of major complication of 7.7 % within 30 days post procedure. Several authors also investigated the safety and feasibility of drug-eluting stent placement for ICAD [24, 25], and albeit yielding better long-term patency rate, they are frequently too stiff to access distal stenosis lesions which might result in a penetration event. Although a relatively higher rate of periprocedural complications was observed in the present study, it may be related to the high grade of stenosis (>70 %) and the complicated stenosis lesions treated in the selected patients.

Atherosclerotic stenosis often involves the origin of a major artery [26]. In this study, the incidence of lesions involving bifurcation or ostium was 63.6 % (28/44). Balloon angioplasty or stenting may result in occlusion of branches due to the atheromatous components squashed into the origin of branches, especially in patients with type D, C, and F locations according to LMA classification, resulting in subsequent infarction. Thus, preprocedural evaluation such as wall imaging of targeted stenosis becomes necessary. Three of the complications of ischemic stroke that occurred in our study were related to occlusion of perforating arteries.

A high rate of ISR is the major limitation of intracranial angioplasty. The ISR rate of Wingspan procedures was reported to range between 7.5 % and 31.2 % [27]. Several factors may contribute to ISR, such as stent thrombogenicity, vascular injury by balloon dilatation, inflammatory reactions of the vessel wall, stent radial force, lesion length, as well as uncontrollable diabetes. Compared with Wingspan, the Enterprise stent exerts less radial force [10], and Zsolt et al. reported satisfactory ISR rates with the Enterprise stent (24.7 % restenosis during a mean 6.9 months of follow-up) compared with Wingspan stent [4]. In the present study, 3 (6.8 %) patients suffered ISR proved by DSA examination, two of whom received further intervention.

The present study has limitations inherent to its retrospective design and relatively small sample size for a 4-year enrollment period, which likely introduce selection bias. Prospective, multicenter, randomized controlled trials with longer-term follow-up and larger sample size are warranted.

## Conclusions

This is a case series study of ICAS treated by Enterprise stent placement. Deployment of an Enterprise stent with undersized balloon angioplasty in the treatment of symptomatic intracranial atherosclerotic stenosis appears safe and effective, with a high technical success rate, relatively low periprocedural complication rate, and favorable outcome during follow-up.
